# Glycosyltransferase-related long non-coding RNA signature predicts the prognosis of colon adenocarcinoma

**DOI:** 10.3389/fonc.2022.954226

**Published:** 2022-09-20

**Authors:** Jiawei Zhang, Yinan Wu, Jiayi Mu, Dijia Xin, Luyao Wang, Yili Fan, Suzhan Zhang, Yang Xu

**Affiliations:** ^1^ Department of Hematology, The Second Affiliated Hospital, Zhejiang University School of Medicine, Hangzhou, China; ^2^ Zhejiang University Cancer Institute, Key Laboratory of Cancer Prevention and Intervention, China National Ministry of Education, The Second Affiliated Hospital, Zhejiang University School of Medicine, Hangzhou, China; ^3^ The Cancer Hospital of the University of Chinese Academy of Sciences(Zhejiang Cancer Hospital), Institute of Basic Medicine and Cancer, Chinese Academy of Sciences, Hangzhou, China; ^4^ Zhejiang Provincial Key Laboratory for Cancer Molecular Cell Biology, Life Sciences Institute, Zhejiang University, Hangzhou, China

**Keywords:** colorectal cancer, glycosyltransferase, lncRNAs, risk model, overall survival

## Abstract

**Purpose:**

Colon adenocarcinoma (COAD) is the most common type of colorectal cancer (CRC) and is associated with poor prognosis. Emerging evidence has demonstrated that glycosylation by long noncoding RNAs (lncRNAs) was associated with COAD progression. To date, however, the prognostic values of glycosyltransferase (GT)-related lncRNAs in COAD are still largely unknown.

**Methods:**

We obtained the expression matrix of mRNAs and lncRNAs in COAD from The Cancer Genome Atlas (TCGA) database. Then, the univariate Cox regression analysis was conducted to identify 33 prognostic GT-related lncRNAs. Subsequently, LASSO and multivariate Cox regression analysis were performed, and 7 of 33 GT-related lncRNAs were selected to conduct a risk model. Gene set enrichment analysis (GSEA) was used to analyze gene signaling pathway enrichment of the risk model. ImmuCellAI, an online tool for estimating the abundance of immune cells, and correlation analysis were used to explore the tumor-infiltrating immune cells in COAD. Finally, the expression levels of seven lncRNAs were detected in colorectal cancer cell lines by reverse transcription-quantitative polymerase chain reaction (RT-qPCR).

**Results:**

A total of 1,140 GT-related lncRNAs were identified, and 7 COAD-specific GT-related lncRNAs (LINC02381, MIR210HG, AC009237.14, AC105219.1, ZEB1-AS1, AC002310.1, and AC020558.2) were selected to conduct a risk model. Patients were divided into high- and low-risk groups based on the median of risk score. The prognosis of the high-risk group was worse than that of the low-risk group, indicating the good reliability and specificity of our risk model. Additionally, a nomogram based on the risk score and clinical traits was built to help clinical decisions. GSEA showed that the risk model was significantly enriched in metabolism-related pathways. Immune infiltration analysis revealed that five types of immune cells were significantly different between groups, and two types of immune cells were negatively correlated with the risk score. Besides, we found that the expression levels of these seven lncRNAs in tumor cells were significantly higher than those in normal cells, which verified the feasibility of the risk model.

**Conclusion:**

The efficient risk model based on seven GT-related lncRNAs has prognostic potential for COAD, which may be novel biomarkers and therapeutic targets for COAD patients.

## Introduction

Colorectal cancer (CRC) is the third leading cause of cancer mortality worldwide. The 5-year overall survival (OS) rate for localized CRC is about 90%, while that for CRC with metastasis is <15% (1, 2). Pathologically, colon adenocarcinoma (COAD) is the most common subtype of CRC (3, 4). At present, the major treatment options for COAD include surgical resection, neoadjuvant chemoradiotherapy, and postoperative chemoradiotherapy (5). Recently, targeted therapy and immunotherapy have shown promising efficacies in patients with COAD (6–8). However, the clinical outcome remains unsatisfactory, largely due to disease heterogeneity arising from the complex interplay between numerous genetic and environmental factors. Thus, it is important to identify novel suitable biomarkers that allow better risk-stratified treatment for COAD patients.

Glycosylation, the most abundant and complex post-translational modification of proteins and lipids, is an enzymatic process regulated by numerous glycosyltransferases (GTs) and glycosidases. In this process, GTs catalyze the transfer of carbohydrate chains to glycoproteins, which is essential for cell adhesion, protein stability, and signal transduction (9). Notably, there are approximately 200 GTs involved in 14 different human protein glycosylation pathways, which are further grouped into N-glycosylation, O-glycosylation, C-mannosylation, and glypiation (10). Aberrant glycosylation signatures on cell surface are closely related to the initiation and development of cancer, primarily through sustaining cell proliferation, enhancing tumor invasion, and immune escape (11–13). More importantly, altered expression of GTs has been shown to correlate with tumorigenesis. For instance, ST6GAL1, a sialyltransferase that catalyzes the transfer of sialic acid to galactose-containing substrates, is highly expressed in CRC and positively associated with microsatellite instability (MSI), BRAF mutations, and mucinous phenotype (14). Fernández et al. confirmed that the upregulation of GCNT3 conferred a better prognosis and improved response to initial therapy in CRC (15). In addition, Gu et al. found that, compared with control mice, the CRC mice showed decreased expression in a subset of GTs, including Mgat1, Mgat2, Mgat3, St6gal1, St3gal4, Fut8, and B4galt1 (16). Cumulative evidence indicates that abnormal expression of GTs seems to be a “hallmark of cancer” and contributes to cancer development.

Long noncoding RNAs (lncRNAs) are RNA species with more than 200 nucleotides (nt) that are not translated to proteins and are involved in the regulation of many essential cellular processes, such as chromatin remodeling, transcription, and post-transcription regulation (17). Studies have demonstrated that lncRNAs play crucial roles in cancer initiation and progression by acting as oncogenes and tumor suppressors (18, 19). They can affect not only the proliferation, migration, and invasion but also energy metabolism of cancer cells (20, 21). Moreover, emerging evidence suggests that lncRNAs play important roles in regulating post-translational modifications of metabolic enzymes, transcription factors, and cancer-associated metabolic pathways (22). Recently, emerging evidence has demonstrated that lncRNAs has been associated with cancer progression *via* regulating the expression level of glycosyltransferases or glycosidases and subsequently influencing the glycosylation pattern. For example, LINC01296 increased the proliferation and metastasis of CRC cells by modulating the O-glycosylated MUC1 *via* PI3K/AKT pathway (23). However, the biological functions of GT-related lncRNAs have rarely been studied.

In this study, we first explored the role of glycosyltransferases in COAD patients from The Cancer Genome Atlas (TCGA) database; then, we screened out seven GT-related lncRNAs with prognostic value, performed a prognostic GT-related lncRNA model, and explored the correlation between the GT-related lncRNA and tumor immune microenvironment (TIM) as well.

## Methods

### Data acquisition

The RNA sequence transcriptome data of COAD patients and relevant clinical information were extracted from The Cancer Genome Atlas (TCGA; https://cancergenome.nih.gov/) database (24). Patients without clinical information were excluded. A total of 429 COAD samples and 37 normal samples were included in the study. The clinical characteristics of the patients are shown in **Table 1**. The corresponding gene set of 210 glycosyltransferases was downloaded from GlycoGene DataBase (GGDB; https://acgg.asia/ggdb2/) (25). The GEO dataset (https://www.ncbi.nlm.nih.gov/geo/) GSE39582, which contains 579 samples, was used to verify the prognostic value of the risk model (26).

**Table 1:** Clinical information of COAD patients in the TCGA database.

Variables	No. of patients	Percentage (%)
Age (years)		
≤60	134	29.2
>60	325	70.8
Gender		
Female	219	47.7
Male	240	52.3
Pathological stage		
I	78	17.0
II	179	39.0
III	125	27.2
IV	64	13.9
Unknown	13	2.8
T stage		
Tis	1	0.2
T1	11	2.4
T2	78	17.0
T3	310	67.5
T4	57	12.4
Unknown	2	0.4
N stage		
N0	272	59.3
N1	104	22.7
N2	81	17.6
Unknown	2	0.4
M stage		
M0	337	73.4
M1	64	13.9
Unknown	58	12.6

### Identification, functional classification, and mutation analysis of differentially expressed GT genes

We used the limma software package in R version (3.6) to identify the differentially expressed GT genes (p value < 0.05 and | log2 (fold change) | > 1.5) between COAD and normal colorectal tissues (27). Gene ontology (GO) and Kyoto Encyclopedia of Genes and Genomes (KEGG) were conducted to analyze the enrichment pathways associated with differentially expressed genes (DEGs). Protein–protein interaction (PPI) network of DEGs was analyzed by the Search Tool for Interaction Genes (STRING) database (28). Single nucleotide variant (SNV) mutation frequency of DEGs was downloaded from GSCALite (http://bioinfo.life.hust.edu.cn/web/GSCALite/) (29). Copy number variation (CNV) mutation frequencies of GT DEGs in COAD patients were extracted from the TCGA database and analyzed by R software.

### GT-related lncRNA identification

We used “limma” R package to identify GT-related lncRNAs (27). Pearson correlation analysis was conducted to detect the association between the expression levels of GT genes and lncRNAs. A total of 1,140 GT-related lncRNAs were identified based on the correlation coefficient >0.3 and p < 0.001.

### Establishing the prognostic risk model of GT-related lncRNAs

To identify GT-related lncRNAs with prognostic value, we conducted the univariate and multivariate COX analysis. We first screened GT-related lncRNAs with potential survival impact on COAD patients based on the standard of p < 0.05 and subsequently incorporated those lncRNAs into LASSO Cox regression analysis and multivariate COX regression analysis (30). The following formula was used to calculate the risk score of each patient: risk score = (coefficient lncRNA_1_ × expression of lncRNA_1_) + (coefficient lncRNA_2_ × expression of lncRNA_2_) +…+ (coefficient lncRNA_n_ × expression of lncRNA_n_). The COAD patients were divided into high- and low-risk groups based on the median value of risk score. R package “timeROC” was used to evaluate the accuracy of risk model (31). Additionally, we used the chi-square test to analyze the relationship between the model and clinical features in order to evaluate the prognostic role of this model.

### Identification of independent prognostic factors

Univariate Cox and multivariate Cox regression analyses were used to evaluate whether the risk score was an independent prognostic factor. Receiver operating characteristic (ROC) curve was made to evaluate the risk score and different clinical characteristics in predicting outcomes (32). Kaplan–Meier (KM) plot was used to analyze the outcome of high- and low-risk groups in subgroups according to clinical characteristics. A p-value <0.05 was considered statistically significant.

### Nomogram and calibration

With “rms” R package, all significantly independent prognostic factors, including age, stage, and risk score were used to build up a nomogram for the 1-, 3-, and 5-year OS (33). The calibration plot was applied to evaluate whether the prediction outcome of the nomogram showed good consistency with practical application.

### Gene set enrichment analyses

With curated gene set (kegg.v7.4.symbols.gmt) and GSEA software (https://www.gsea-msigdb.org/gsea/login.jsp), GSEA analysis was conducted in the COAD cohort to explore the significantly enriched pathways between the high- and low-risk groups based on the criterion: p<0.05 and false discovery rate (FDR) <0.25 (34, 35).

### Analysis of immune cell characteristics and immune-specific gene expression in the risk model

Immune Cell Abundance Identifier (ImmuCellAI) is an analytical tool that provides the quantitative infiltration of immune cells and predict the response of immune checkpoint blockade (ICB) therapy by using gene expression matrix data (36). We estimated the relative abundance of immune cell subtypes for each sample based on gene expression data. The abundance of immune cell subtypes and response to ICB therapy were compared between the high- and low-risk groups based on p < 0.05. The correlation between risk scores and immune infiltration was calculated by Pearson correlation analysis. Besides, the expression of immune-specific genes was compared between high- and low-risk groups.

### Cell culture and reverse transcription and quantitative PCR analysis

The COAD cell lines (HCT116, DLD1, and HT-29) and normal human colon mucosal epithelial cell line NCM460 were acquired from the American Type Culture Collection (ATCC, USA). All cells were cultured in Roswell Park Memorial Institute (RPMI)-1640 medium (Gibco, CA, USA) with 10% fetal bovine serum (FBS, Gibco, CA, USA), 100 U/ml penicillin, and 100 mg/ml streptomycin. To evaluate the expression levels of GT-related lncRNAs, we used RNA Trizol reagent (Invitrogen, USA) to extract total cellular RNA, which were then reverse transcribed to cDNA using PrimeScript™ RT reagent Kit (Takara, Japan). Real-time fluorescent quantitative PCR was performed by using SYBR Green Master Mix (Yeasen, China). The β-actin mRNA was chosen as an endogenous control. The expression level of GT-related lncRNAs was analyzed using 2^−ΔΔCT^. Each PCR reaction was performed in triplicate. The primer sequences are listed in **Supplementary Table S1**.

### Statistical analysis

The data analysis of this study was performed by R software (version 3.6; https://www.r-project.org/) and GraphPad Prism 8 software. One-way analysis of variance (ANOVA) was used to compare the expression level of GT in 429 COAD tissues and 37 normal tissues. The two-tailed t-test was performed to analyze continuous variables between the two subgroups, and the Chi-square test was conducted to evaluate categorical data. The DEGs co-expression network and GT-lncRNA-mRNA co-expression network were conducted by the Cytoscape software (version 3.6.0; https://cytoscape.org/). The Kaplan–Meier method was used to compare the OS of each group based on the median value, and log-rank tests were applied to evaluate the significance of differences. The RT-qPCR results were analyzed using one-way ANOVA, a p < 0.05 on both sides was considered statistically significant.

## Results

### Differentially expressed GTs in the COAD and normal colorectal tissues

**Figure 1:**
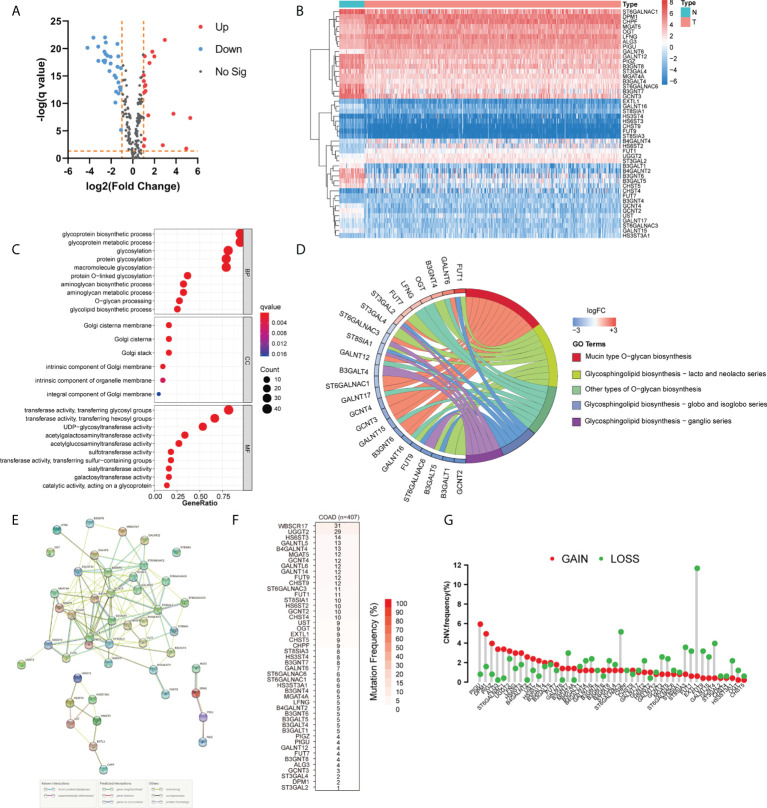
Landscape of genetic and expression variation of glycosyltransferase genes in COAD. **(A, B)** Volcano and heatmap visually showed differentially expressed genes between normal and COAD. **(C, D)** GO and KEGG analysis of DEGs. **(E)** Protein–protein interaction (PPI) network showing the interaction between DEGs among glycosyltransferases. **(F)** The SNV mutation frequency of DEGs in the COAD cohort. **(G)** The CNV variation frequency of DEGs in COAD. The height of the column represented the alteration frequency. N, normal tissues; T, tumor; BP, biological process; CC, cellular component; MF, molecular function; FC, fold change.

To identify the differentially expressed GTs and explore the biological functions of GTs in COAD, we used the TCGA database to analyze 429 COAD and 37 normal tissues. We uncovered 46 differentially expressed GTs (18 upregulated and 28 downregulated, **Figures 1A, B**; **Supplementary Table S2**). The GO analyses revealed that these DEGs were most enriched in glycoprotein biosynthetic and metabolic processes (**Figures 1C, D**). KEGG analysis showed that 46 DEGs mainly involved in the mucin type O-glycan biosynthesis and glycosphingolipid biosynthesis including lacto and neolacto series, globo and isoglobo series, and ganglio series. We also clarified the relationship between these differentially expressed GTs *via* STRING database (**Figure 1E**). The incidence of single nucleotide variant (SNV) and copy number variations (CNVs) of differentially expressed GTs were analyzed in COAD. As shown in **Figure 1F**, SNV mutations were found in all 46 DEGs, among which WBSCR17 had the highest mutation frequency (31%), followed by UGGT2 (29%) and HS6ST3 (14%). We also found that CNV alterations were common in DEGs. Nearly half of the DEGs had copy number deletion, while the CNV amplification frequencies of PIGU, DPM1, PIGZ, ALG3, ST6GALNAC1, UGGT2, LFNG, HS6ST3, B4GALNT2, UST, HS3ST4, and GALNT6 were widespread (**Figure 1G**).

### Identification of GT-related lncRNAs with prognostic value

From the TCGA database, we identified 1,140 GT-related lncRNAs. We further combined these lncRNAs with COAD survival data in univariate COX regression analysis to identify 33 GT-related lncRNAs with significant prognostic value. As a result, 32 GT-related lncRNAs were found to be associated with increased cancer risk (p < 0.05 and HR > 1), while only 1 lncRNA, AC124067.4, was a protective factor for COAD (p < 0.05 and HR < 1) (**Table 2**; **Supplementary Figure S1A**). In correlation analysis, almost all prognostic lncRNAs have a weak to moderate correlation with other lncRNAs, whereas LINC00174 and AC026471.4, and LINC00174 and AC107375.1 had the strongest correlation (r =0.58, and 0.57, respectively) (**Supplementary Figure S1B**).

**Table 2:** Thirty-three GT-related lncRNA associated with prognosis in patients with COAD.

Gene	HR	HR.95L	HR.95H	p-value
LINC02381	1.25242	1.068814	1.467567	0.00539
LBX2-AS1	1.0964	1.004362	1.196872	0.039662
AC005083.1	1.07699	1.00173	1.157904	0.044778
AC002310.1	1.942366	1.491101	2.5302	8.58E-07
AC068580.3	1.394748	1.0308	1.887196	0.031037
AC068870.2	1.220003	1.075456	1.383978	0.001998
AL354707.1	1.163373	1.016503	1.331464	0.027972
AC073869.1	1.096303	1.011486	1.188232	0.025224
AC011462.4	1.669805	1.230889	2.26523	0.000984
PCAT6	1.251075	1.121586	1.395514	5.86E-05
AL035587.1	1.467435	1.08192	1.990319	0.013651
LINC00957	1.388302	1.056806	1.823782	0.018429
AL162586.1	1.529439	1.205452	1.940502	0.000468
AL392172.1	1.203027	1.032544	1.401658	0.017754
LINC01011	1.713264	1.228365	2.389578	0.001516
ZEB1-AS1	2.353207	1.634573	3.387787	4.17E-06
AC020558.2	1.618302	1.119727	2.338874	0.010414
AC007128.1	1.538297	1.095726	2.159627	0.012843
RPARP-AS1	1.223619	1.024764	1.461062	0.025727
AC107375.1	1.272858	1.004496	1.612915	0.045815
AC105219.1	1.15947	1.034716	1.299265	0.010848
AL451050.2	1.537851	1.085384	2.178939	0.015485
AL161729.4	1.368832	1.105511	1.694872	0.003975
MIR210HG	1.161189	1.071914	1.257899	0.000251
LINC00174	1.426197	1.124088	1.809501	0.003466
AC063948.1	1.869623	1.322202	2.643689	0.0004
AC026471.4	1.172591	1.043594	1.317534	0.007416
AL354836.1	1.108125	1.013558	1.211514	0.024079
LINC01138	1.632056	1.164118	2.28809	0.00449
AC005261.3	1.209838	1.018099	1.437687	0.030487
AC124067.4	0.964575	0.930648	0.999738	0.048344
AC009237.14	1.1831	1.079035	1.297201	0.000345
AL450326.1	1.471468	1.003744	2.157144	0.047804

### Generation of a GT-related lncRNA risk model

**Figure 2:**
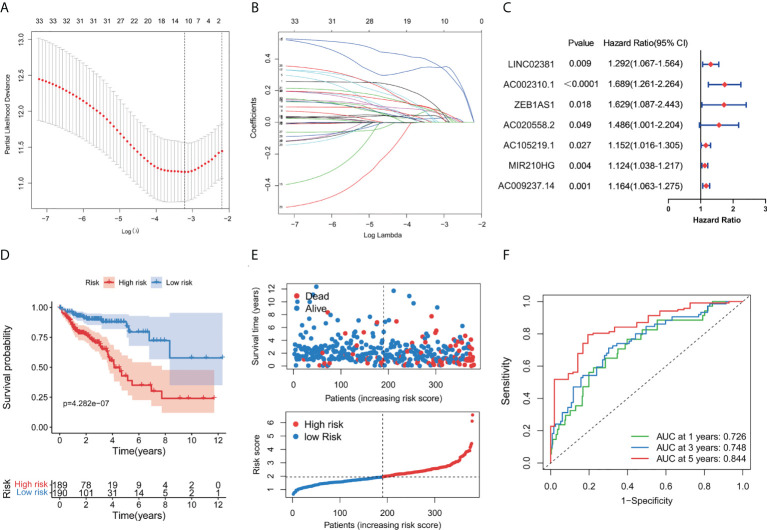
Construction of a prognostic GT-related lncRNA risk model. **(A)** Screening of optimal parameters (lambda) in the LASSO regression model. **(B)** LASSO coefficient profiles of 11 candidate GT-related lncRNAs. **(C)** Forest map of seven GT-related lncRNAs significantly correlated with outcome and identified by multivariate cox regression. **(D)** Kaplan–Meier curve for the OS of COAD patients in the high- and low-risk group. **(E)** Distribution of survival status and risk score in the patient cohort. **(F)** ROC curves at 1, 3, and 5 years in COAD patients.

**Figure 3:**
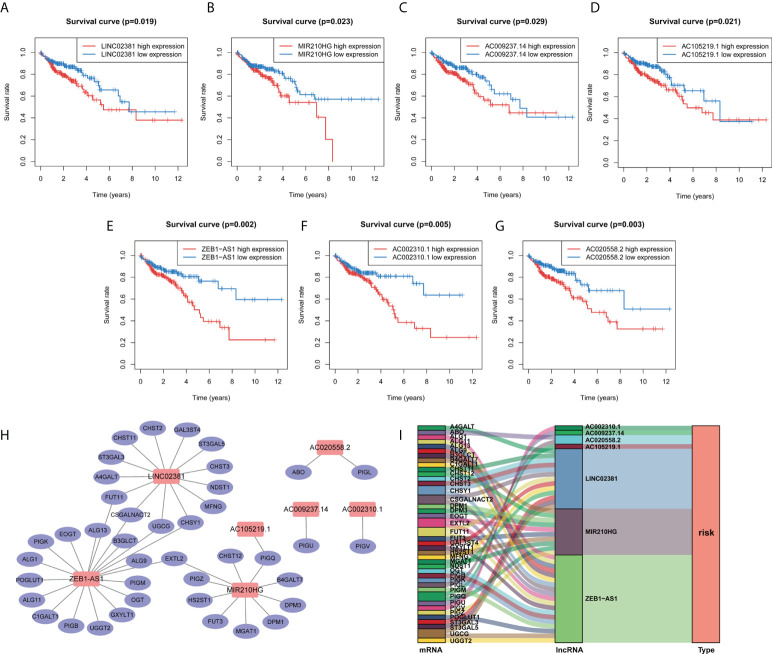
Kaplan–Meier survival curve of the selected GT-related lncRNAs and co-expression network between lncRNA and mRNA. **(A–G)** The overall survival curve of LINC02381, AC002310.1, ZEB1AS1, AC020558.2, AC105219.1, MIR210HG, and AC009237.14 of COAD patients in the high- and low-risk groups. **(H)** Co-expression network of GT genes and prognostic lncRNAs. Red nodes represent GT-related lncRNAs, while blue nodes represent GT genes. **(I)** The relationships among GT genes, GT-related lncRNAs, and risk type in the Sankey diagram.

To better clarify the prognostic potential of GT-related lncRNAs, we conducted the LASSO and multivariate COX analysis on 33 GT-related lncRNAs (**Figures 2A, B**). Ultimately, seven lncRNAs, namely, LINC02381, AC002310.1, ZEB1AS1, AC020558.2, AC105219.1, MIR210HG, and AC009237.14, were identified to construct the prediction model (**Figure 2C**). The prognostic risk model was established based on the following formulation: risk score = (0.2552 × expression value of LINC02381) + (0.5370 × expression value of AC002310.1) + (0.4937 × expression value of ZEB1AS1) + (0.3940 × expression value of AC020558.2) + 0.1416 × expression value of AC105219.1) + (0.1187 × expression value of MIR210HG) + (0.1524 × expression value of AC009237.14). The risk score of patients was calculated, and COAD patients were divided into high- and low-risk groups according to the median risk score. The OS in high-risk patients was significantly lower than that in low-risk patients (p < 0.001, **Figure 2D**). The distribution of the risk score and survival status are shown in **Figure 2E**. The risk score had a good prognostic predictive ability with the area under the ROC curve (AUC) values at 1, 3, and 5 years of 0.726, 0.748, and 0.844, respectively (**Figure 2F**). Furthermore, we investigated the prognostic implication of the expression of lncRNAs. The OS of patients in the high-expression group of seven lncRNAs was significantly lower than that in the low-expression group (all p < 0.05, **Figures 3A–G**). We conducted a lncRNA–mRNA co-expression network to visualize the relationship among seven prognostic lncRNAs and GTs (**Figure 3H**). According to the risk score, a Sanky diagram was produced to exhibit the association among GTs, GT-related lncRNA, and outcome types of lncRNAs, indicating a single lncRNA corresponding to one or more mRNA, and these seven GT-related lncRNAs were all risk factors for a poor prognosis (**Figure 3I**). To verify the prognostic value of the risk model, we acquired the GSE39582 dataset from GEO database and then extracted the expression levels of seven GT-related lncRNAs and the survival data of patients. The results showed that patients with low-risk score had a better prognosis compared to those with high-risk score (**Supplementary Figure S2**).

### Correlation between risk model and clinicopathological features

**Figure 4:**
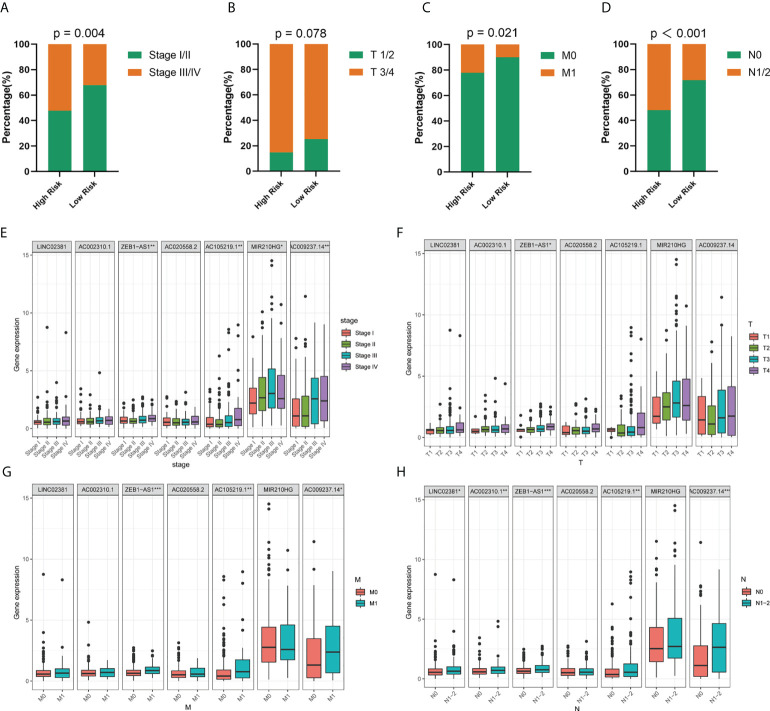
Correlation between risk model and clinicopathological factors. **(A–D)** Distribution of stage I/II and III/IV, T1/2 and T3/4, M0 and M1, and N0 and N1/2 tumors between high- and low-risk group. **(E–H)** Expression levels of seven prognostic GT-related lncRNAs in T, M, N, and S stage groups. *p < 0.05, **p < 0.01, and ***p < 0.001.

In the next step, we explored the relationship between the risk model and clinical variables. Significant differences were found between high- and low-risk groups in the pathological stage, T stage, M stage, and N stage, whereas the two groups showed no significant differences in age and gender (**Figures 4A–D**; **Supplementary Figure S3**). The high-risk group underwent significant upstaging compared with those in the low-risk group (52.2% *vs*. 2.1%, p=0.004). With the respect to the distribution of the American Joint Committee on Cancer (AJCC) stage, the high-risk group also comprised much poorer AJCC stage (T3/4, M1, and N1/2) than the low-risk group (85.1% *vs*. 74.7%, p = 0.078; 14.9% *vs*. 9.9%, p = 0.021; 51.9% *vs*. 28.4%, p < 0.001, respectively). Next, we performed an in-depth analysis of the correlation between seven lncRNAs and clinical variables (**Figures 4E–H**). In the pathological stage, the expression level of four lncRNAs (ZEB-AS1, AC105219.1, MIR210HG, and AC009237.14) was gradually elevated as the tumor stage progressed. For T stage, only one lncRNA was identified with significant differences across subgroups. Regarding M and N stage, three and five lncRNAs, respectively, were found with different expressions among different subgroups. Interestingly, we noticed that the expression level of ZEB1-AS1 was significantly increased with the progression of all stages (T, M, N, and S), suggesting that this lncRNA might be a core prognostic factor in COAD.

### Independent prognostic role of the GT-related lncRNA signature

**Figure 5:**
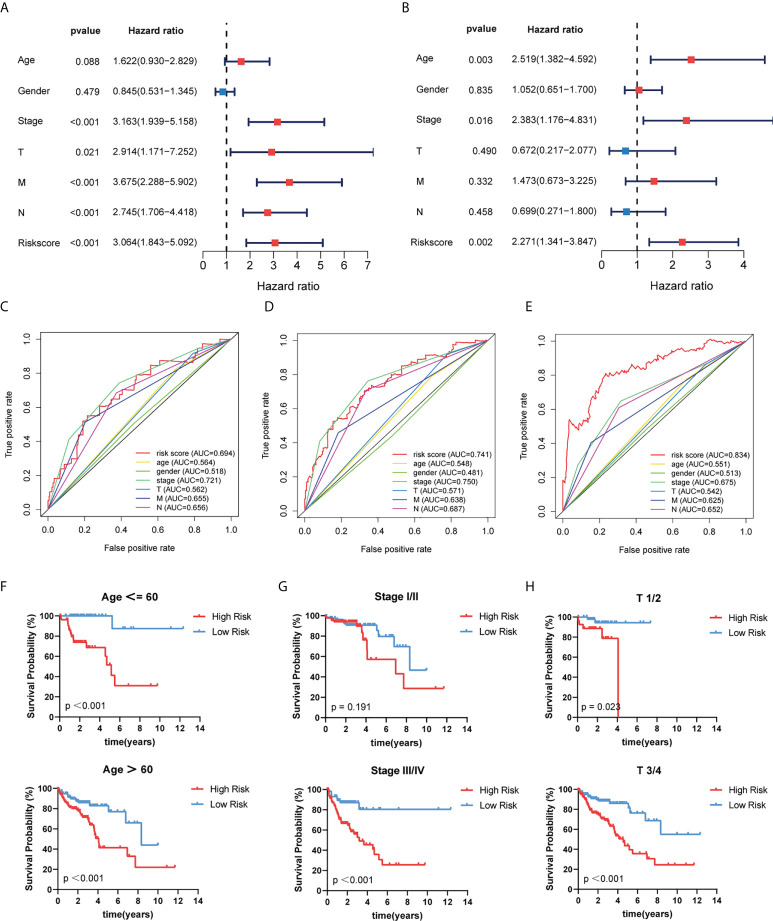
Independent prognostic role of risk model signature. **(A, B)** Univariate and multivariate Cox regression analyses of risk scores and clinical characteristics. **(C–E)** Determination of the area under the ROC curve (AUC) of the risk score and clinical features based on the ROC curve. **(F–H)** The Kaplan–Meier curve shows the prognostic value of the risk model for COAD patients categorized by age, stage, and T stage.

We used univariate and multivariate COX regression analyses to evaluate whether the risk model had an independent prognosis value. The results of univariate and multivariate analysis revealed that age (HR=1.622 and 2.519, respectively; 95%CI, 0.930–2.829 and 1.382–4.592, respectively; p = 0.088 and 0.003, respectively), stage (HR=3.163 and 2.383, respectively; 95%CI, 1.939–5.158 and 1.176–4.831, respectively; p < 0.001 and 0.016, respectively), and risk score (HR=3.064 and 2.271, respectively; 95%CI, 1.843–5.092 and 1.341–3.847, respectively; p < 0.001 and 0.002, respectively) can be independent prognostic factors (**Figures 5A, B**). Time-dependent ROC curves were conducted to evaluate the prognostic and predictive ability of clinical features and risk score (**Figures 5C–E**). Results showed that the AUC value of risk scores at 1, 3, and 5 years was 0.694, 0.741, and 0.834, respectively. More importantly, the risk score had higher precision compared with other predictors. As we all know, age, gender, and clinical stage are identified as independent prognostic characteristics of COAD patients. Then, patients were stratified according to age (≤60 and >60), gender (female and male), stage (I/II and III/IV), T stage (T1/2 and T3/4), M stage (M0 and M1), and N stage (N0 and N1/2) (**Figures 5F–H**; **Supplementary Figure S4**). Among the subgroups, patients in the high-risk group exhibited much poorer survivability than those in the low-risk group.

### Construction of GT-related lncRNAs nomogram based on the prognostic model and clinical characters

**Figure 6:**
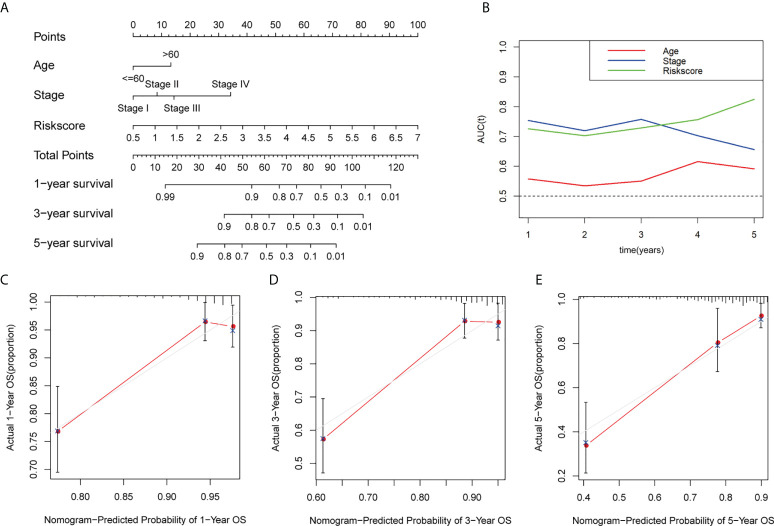
Construction and calibration of the nomogram. **(A)** Construction of a nomogram based on age, stage, and risk score as independent prognostic factors. **(B)** Time-dependent ROC analysis based on the nomogram and clinical characteristics. **(C–E)** The calibration plot for internal validation of the nomogram within 1, 3, and 5 years, respectively. AUC, the area under the ROC curve.

To evaluate the clinical characters and risk model for colorectal cancer prognosis, we integrated age, stage, and risk score to build a nomogram (**Figure 6A**). Additionally, a time-dependent ROC analysis was performed to evaluate whether risk score and clinical traits were capable for survival prediction (**Figure 6B**). The AUC value of risk score in time of 5-year follow-up was 0.726, 0.703, 0.729, 0.757, and 0.825, respectively. The AUC value of stage in time of 5-year follow-up was 0.755, 0.720, 0.758, 0.704, and 0.656, respectively. For age, the average AUC was below 0.6 during a follow-up of 5 years. These results showed that risk score exhibited much more powerful capacity of survival prediction compared with other clinical traits. Besides, calibration plots in 1, 3, and 5 years are shown in **Figures 6C–E**, and it revealed that the observed *vs*. predicted rates of 1-, 3-, and 5-year overall survival were in perfect concordance.

### GSEA enrichment analysis and immune cell infiltration in GT-related lncRNA signature

**Figure 7:**
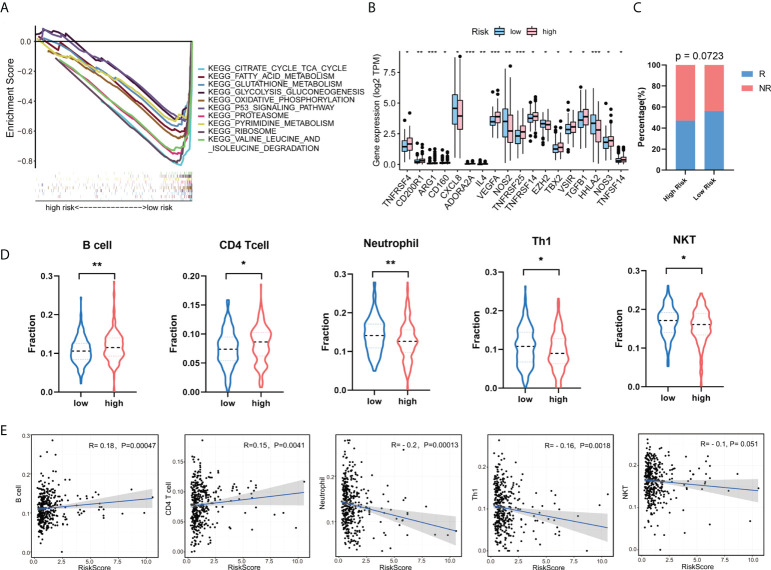
Gene set enrichment analysis (GSEA) and the correlation between risk model and tumor-infiltrating immune cells. **(A)** Enrichment analysis of signaling pathways in the risk model. **(B)** The expression levels of immune-related genes between the high- and low-risk groups. **(C)** The response of immune checkpoint blockade (ICB) therapy between the high- and low-risk group. R, response; NR, no response. **(D)** The fraction of tumor immune infiltrating cells in the high- and low-risk group. **(E)** Correlation of risk score with five tumor-infiltrating immune cell subtypes. *p < 0.05, **p < 0.01, ***p < 0.001. Th1, T helper 1; NKT, natural killer T.

To further explore the abnormally activated signal pathways between the high- and low-risk groups, we performed GSEA enrichment analysis. The results showed that the low-risk group was involved in several important pathways, including citrate cycle TCA cycle, fatty acid metabolism, glutathione metabolism, glycolysis gluconeogenesis, oxidative phosphorylation, P53 signaling pathway, proteasome, pyrimidine metabolism, ribosome, and valine leucine and isoleucine degradation (**Figure 7A**). Given the important role of tumor immune microenvironment (TIM), we looked into the expression levels of immune-related genes between the high- and low-risk score groups. The result showed that CD200R1, ARG1, CD160, ADORA2A, IL4, VEGFA, TNFRSF4, TNFRSF14, TNFRSF25, TBX2, VSIR, TGFB1, and NOS3 are increased, while CXCL8, NOS2, EZH2, and HHLA2 are decreased in the high-risk group compared with the low-risk group (**Figure 7B**). In addition, Pearson correlation analysis was conducted to analyzed the correlation between expression levels of immune-related genes and seven GT-related lncRNAs (**Supplementary Table S3**). The results turn out that AC020558.2, AC105219.1, and MIR210HG were positively correlated with TNFRSF25 and TNFRSF14, which are immune suppression genes, while LINC02381, AC002310.1, and ZEB1-AS1 were negatively associated with immune response gene NOS2 and HHLA2. ImmuCellAI is an online tool to estimate the response of immune checkpoint blockade (ICB) therapy and the infiltration of immune cells based on the gene expression dataset (36). We used ImmuCellAI to predict the response of ICB therapy between the high- and low-risk groups. The result revealed that patients in the low-risk group tend to have a better response to ICB therapy than those in the high-risk group, even though the difference was not significant (p=0.0723, **Figure 7C**). The abundance of immune cells with significant differences between the high- and low-risk groups is shown in **Figure 7D**. The levels of B cell and CD4+ T cell in the high-risk group were higher than those in the low-risk group, while the levels of T-helper 1 cells (Th1), neutrophil, and natural killer T (NKT) in the high-risk group were lower than those in the low-risk group (p<0.05). To determine the correlation between the risk score and tumor-infiltrating immune cells, we found that the risk score was positively correlated with B cell and CD4+ T cell and negatively correlated with Th1 and neutrophil (p<0.05) (**Figure 7E**). Taken together, these data suggest that our risk model can distinguish different characteristics of tumor immune cells in COAD.

### Expression levels of GT-related lncRNAs in COAD cell lines

**Figure 8:**
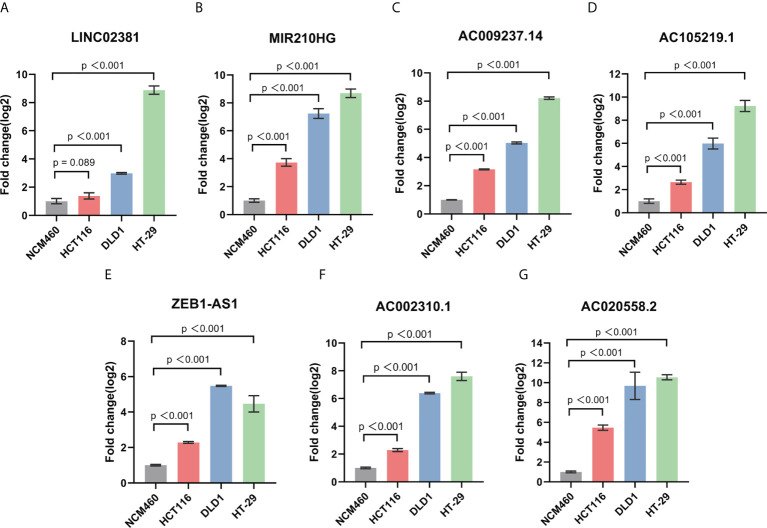
The expression level of LINC02381 **(A)**, MIR210HG **(B)**, AC009237.14 **(C)**, AC105219.1 **(D)**, ZEB1-AS1 **(E)**, AC002310.1 **(F)**, AC020558.2 **(G)** between normal cell line NCM460 and HCT116, DLD1, and HT-29 CRC cell lines.

To further verify our results, we used RT-qPCR assay to analyze the expression level of seven GT-related lncRNAs in COAD cell lines (**Figures 8A–G**). Three human COAD cell lines, namely, HCT116, DLD1, and HT-29, were used, and NCM460, a normal human colon mucosal epithelial cell line, was used as control. Almost all seven prognostic GT-related lncRNAs were upregulated in COAD cell lines, indicating that these GT-related lncRNAs play key roles during progression of COAD. The results were generally consistent with our work.

## Discussion

COAD, as the most frequent type of colorectal cancer, have been widely investigated in terms of its rapid development and treatment. The circulating tumor DNA, tumor-infiltrating immune cells, and microsatellite status have emerged as promising biomarkers for the diagnosis and treatment of CRC (37–39). As an important epigenetic player in cancer pathogenesis, lncRNA together with N6-methyladenosine (m (6)A) RNA methylation has been shown to predict treatment response and disease prognosis in CRC patients (40, 41). Epigenetic modifications have gained much attention in the research on the development of CRC, which is regarded as a diagnostic and predictive biomarker of CRC patients (42, 43). Protein glycosylation is the most common post-translational modification, in which GTs catalyze the formation of glycoproteins (44). Thus, dysregulation of GTs may contribute to aberrant glycosylation, leading to tumorigenesis. However, the role of GT-related lncRNAs in COAD remains poorly defined. For this purpose, it is necessary to construct a useful risk model based on the GT-related lncRNAs.

In this study, we acquired RNA-sequencing data and clinical information of COAD from the TCGA database and extracted 210 GT genes from the GlycoGene DataBase. First, we identified 46 differentially expressed GT genes and explore their mutation frequency in COAD. Then, we screened 1,140 GT-related lncRNAs based on the co-expression of lncRNAs and GTs. Subsequently, seven prognostic GT-related lncRNAs (LINC02381, AC002310.1, ZEB1AS1, AC020558.2, AC105219.1, MIR210HG, and AC009237.14) were identified through LASSO and Cox regression analysis (30). The risk signature was determined based on the seven GT-related lncRNAs, which stratified COAD patients into two high- and low-risk groups. Survival analysis revealed that patients in the high-risk group had a much worse prognosis. Moreover, patients with high expression levels of seven GT-related lncRNAs turned out to have poorer survivability, suggesting these lncRNAs as risk factors in COAD prognosis. Next, we performed univariate and multivariate Cox regression analyses to identify whether risk score can be an independent prognostic factor. In subgroup analyses, patients in the high-risk subgroup had poorer clinical phenotypes compared with those in the low-risk subgroup, indicating that our risk signature could affect the progression of COAD patients. A nomogram based on risk score, age, and stage was built to predict the prognosis of patients over 1, 3, and 5 years, which showed good performance in clinical practice. Moreover, a time-dependent ROC analysis was conducted to validate the accuracy of the risk model. Compared with the AUC value of age and stage, risk score presented more powerful capacity in survival prediction. These results turned out that our prognostic risk signature can be used as a potential predictor in COAD prognosis.

Currently, only LINC02381, ZEB1AS1, and MIR210HG have been studied extensively. Huang et al. found that LINC02381 was upregulated in breast cancer, and knockdown of LINC02381 impaired the malignant phenotypes of breast cancer cells, including cell proliferation, migration, and invasion (45). Moreover, LINC02381 plays an oncogenic role in cervical cancer and osteosarcoma (46, 47). ZEB1AS1 is a famous cancer-related lncRNA, and its overexpression is widely found in various cancers, including colorectal cancer (48). Lv et al. demonstrated that ZEB1AS1 was increased in CRC, and it promoted CRC cell proliferation *via* mediating Wnt/β-catenin signaling (49). CRC patients with high ZEB1AS1 expression led to a poor prognosis and lower OS rate than those with low ZEB1AS1 expression, suggesting that ZEB1-AS1 is a promising biomarker in predicting clinical outcomes (50, 51). Studies about MIR210HG in tumorigenesis have been widely reported. In pancreatic cancer, MIR210HG acted as oncogenic regulator and promoted pancreatic cell proliferation and migration (52). In addition to pancreatic cancer, MIR210HG can also promote various cancer types, such as breast cancer, gastric cancer, and ovarian cancer (53–55). Additionally, previous studies have identified seven GT-related lncRNAs as prognostic factors in colon cancer patients. For example, ZEB1-AS1, LINC02381, AC105219.1, and AC002310.1 were identified as independent prognostic factors in predicting survival in colon cancer patients (56). MIR210HG and AC009237.14 also play an important role in predicting prognosis in colon cancer patients (57). This evidence make our results more reliable, suggesting that our GT-related lncRNA signature has a promising potential for predicting COAD prognosis.

To further explore the potential biological mechanism of our prognostic risk signature, we further used GSEA to determine the enriched signaling pathways. The results showed that the prognostic risk signature was mainly enriched in metabolism-related signaling pathways. Abnormal metabolism plays a critical functional role in colorectal cancer development and progression; thus, targeting key metabolic factors might be a potential treatment in colorectal cancer. Ag120 (ivosidenib), an inhibitor for glutamine uptaking protein ASCT/SLC1A5, was found to block glutamine transport and metabolism, thus leading to impaired cell proliferation, increased autophagy, and oxidative stress (58). Besides, our prognostic risk model was also enriched in P53 pathway signaling. P53 was encoded by TP53 gene, and mutations of TP53 have been associated with poor prognosis in various cancer types, including colorectal cancers (59). In advanced CRC patients with distant metastases, the TP53 mutation rate reached 80% (60). The specific mechanism by GT-related lncRNAs participating in the regulation of COAD biological process requires further *in vivo* and *in vitro* experimentation.

In addition, we compared immune-related genes between high- and low-risk groups. Interestingly, several immunosuppression-related genes were significantly higher expressed in the high-risk group, including CD200R1, ARG1, TNFRSF4, TNFRSF14, and VSIR. These genes have been reported to be associated with the inhibition of T-cell responses and cell apoptosis (61–65). Besides, several tumor-related genes, like VEGFA, TBX2, and TGFB1, were also enriched in the high-risk group (66–68). These findings suggested that patients in the high-risk group might have a poorer prognosis compared with those in the low-risk group. Recently, studies about the role of lncRNAs in TIM have been widely reported. The prognostic value of immune-related lncRNAs has been found in various cancers. Currently, a prognostic model has been conducted based on eight immune-related lncRNAs pairs that can effectively predict the prognosis of patients with COAD (69). Here, we performed an in-depth analysis of the relationship between the risk model and the distribution of immune-infiltrating cells by ImmuCellAI. We found that the high- and low-risk groups significantly distinguished the characteristics of B cells, CD4+ T cells, Th1, neutrophils, and NKT cells, among which B cells and CD4+ T cells exhibited a higher degree of infiltration in the high-risk group than in the low-risk group. Alternatively, Th1, neutrophils, and NKT cells exhibited a higher degree of infiltration in the low-risk group than in the high-risk group. In this study, the results of correlation between risk score and immune-infiltrating cells showed that the risk score was positively correlated with B cells and CD4+ T cells and negatively correlated with Th1 and neutrophils. Altogether, our results showed that the risk model could evaluate the tumor infiltrating-immune cells to analyze the tumor immune characteristics, thereby determining the prognosis of patients with COAD.

To further validate the accuracy of our model, we employed GSE39582 as the validation set, and the same results were obtained, suggesting that our risk model had a good prognostic predictive ability in COAD patients. Moreover, we conducted RT-qPCR assay to analyze the expression level of seven GT-related lncRNAs in COAD cell lines. The expression of seven GT-related lncRNAs were significantly upregulated in COAD cells than that in the control cell, which were consistent with our model. Combined with **Figures 3C** and **4A–G**, high expression levels of seven GT-related lncRNAs were associated with worse survival, HR>1, indicating that they might play a role as cancer‐promoting genes, which were consistent with our risk model.

## Conclusion

The risk model based on seven GT-related lncRNAs has independent prognostic value and high reliability in predicting the prognosis of patients with colorectal cancer. Owing to the important role of lncRNAs in the cellular process, these GT-related lncRNAs might be promising biomarkers and targets for CRC diagnosis and treatment, respectively. Moreover, the risk model for COAD was transformed into a nomogram, providing a convenient tool for clinical practice and new sights for a better understanding of the mechanism of immune cell-specific genes and tumor infiltrating-immune cells in cancer regulation.

## Data availability statement

The original contributions presented in the study are included in the article/**Supplementary Material**. Further inquiries can be directed to the corresponding authors.

## Author contributions

JZ, YW, and YX designed the study. JZ, YW, and JM acquired the data and performed data analysis and interpretation. JZ and YW drafted the manuscript. DX, LW, and YF provided suggestion to improve it. SZ and YX critically revised it for important intellectual content. All authors contributed to the article and approved the submitted version.

## Funding

This study was supported by the Natural Science Foundation of Zhejiang Province of China (LY21H080005) and National Natural Science Foundation of China (81572920).

## Conflict of interest

The authors declare that the research was conducted in the absence of any commercial or financial relationships that could be construed as a potential conflict of interest.

## Publisher’s note

All claims expressed in this article are solely those of the authors and do not necessarily represent those of their affiliated organizations, or those of the publisher, the editors and the reviewers. Any product that may be evaluated in this article, or claim that may be made by its manufacturer, is not guaranteed or endorsed by the publisher.
