# Supplementary material for: Glycosyltransferase-related long non-coding RNA signature predicts the prognosis of colon adenocarcinoma
Source: Front Oncol. 2022 Sep 20;12:954226. doi: 10.3389/fonc.2022.954226 (PMC9530784; doi:10.3389/fonc.2022.954226)
Supplement: Supplementary file 1 [file DataSheet_1.docx]

Supplementary Material

| lncRNA | Primer |
| --- | --- |
| LINC02878-F | AGATGCGGCGGATCATAAGG |
| LINC02878-R | AATCCTGCACAAGAGCGTCA |
| MIR210HG-F | GCAGGCACAGGTGTGGTCATATC |
| MIR210HG-R | AGGCAGGCTCAGCAGACAGG |
| AC009237.14-F | GGTCTGTGATTCTGCTGATGG |
| AC009237.14-R | CCCCTGGAGTCTTTCTTTGA |
| AC105219.1-F | AGATGCGGCGGATCATAAGG |
| AC105219.1-R | AATCCTGCACAAGAGCGTCA |
| ZEB1-AS1-F | TCGCCTACCGAATCAGGTCA |
| ZEB1-AS1-R | CTTAGCAGGGAGGTACAGGC |
| AC002310.1-F | TCCCCATTCCGATTCCATCC |
| AC002310.1-R | CTACGGTCTCTTACCGTGCC |
| AC020558.2-F | ACCACAGCACTCTTCATGGG |
| AC020558.2-R | GCAAGGACCCTCTGAATGCT |
| B-actin-F | GATCATTGCTCCTCCTGAGC |
| B-actin-R | ACTCCTGCTTGCTGATCCAC |

**Supplementary Table 1.** The lncRNAs primer sequences used in this study.

| Gene | NormalMean | TumorMean | LogFC | pValue | fdr |
| --- | --- | --- | --- | --- | --- |
| CHST4 | 0.0379966 | 1.5166765 | 5.3188994 | 2.33E-08 | 4.56E-08 |
| HS3ST4 | 0.0084875 | 0.2623443 | 4.9499752 | 0.0154398 | 0.0177457 |
| HS6ST2 | 0.2913438 | 3.9448132 | 3.7591626 | 3.31E-09 | 7.92E-09 |
| FUT1 | 0.2990184 | 2.326272 | 2.9597139 | 4.55E-24 | 2.69E-22 |
| B4GALNT4 | 0.4772378 | 3.2572706 | 2.7708833 | 0.0036428 | 0.0043566 |
| GALNT6 | 3.076224 | 12.268489 | 1.9957252 | 2.30E-20 | 2.71E-19 |
| CHPF | 15.645143 | 54.430732 | 1.7987066 | 1.69E-21 | 3.74E-20 |
| UGGT2 | 1.2757793 | 3.7056116 | 1.5383329 | 5.04E-19 | 4.25E-18 |
| B3GNT4 | 0.1207911 | 0.3253078 | 1.4292907 | 6.85E-09 | 1.52E-08 |
| MGAT5 | 11.133764 | 25.154247 | 1.1758606 | 1.38E-13 | 5.44E-13 |
| OGT | 9.2884604 | 20.964452 | 1.1744338 | 1.04E-14 | 4.72E-14 |
| DPM1 | 23.348656 | 50.478735 | 1.1123363 | 1.56E-14 | 6.58E-14 |
| ALG3 | 11.715092 | 25.18939 | 1.1044479 | 1.86E-20 | 2.53E-19 |
| HS3ST3A1 | 0.195511 | 0.4154661 | 1.0874813 | 0.0002514 | 0.0003408 |
| LFNG | 17.982535 | 37.516282 | 1.0609204 | 2.66E-13 | 1.00E-12 |
| PIGU | 11.347797 | 23.117118 | 1.0265493 | 1.78E-15 | 8.50E-15 |
| FUT7 | 0.1412504 | 0.2875084 | 1.0253497 | 0.0048443 | 0.0057547 |
| ST3GAL2 | 2.1172101 | 4.2883457 | 1.0182568 | 1.22E-16 | 7.42E-16 |
| ST3GAL4 | 17.81751 | 8.3524712 | -1.093021 | 4.36E-06 | 6.89E-06 |
| MGAT4A | 14.330513 | 6.6750391 | -1.102242 | 5.42E-18 | 3.83E-17 |
| ST6GALNAC3 | 0.6217709 | 0.2808059 | -1.14681 | 8.20E-14 | 3.30E-13 |
| UST | 1.5357021 | 0.6734774 | -1.189197 | 2.76E-14 | 1.13E-13 |
| HS6ST3 | 0.0696342 | 0.0278955 | -1.319765 | 3.98E-13 | 1.47E-12 |
| ST8SIA1 | 0.294418 | 0.1170195 | -1.331117 | 3.16E-15 | 1.47E-14 |
| GALNT12 | 35.940265 | 14.156008 | -1.344187 | 2.03E-20 | 2.57E-19 |
| B3GNT8 | 34.243968 | 12.828928 | -1.416449 | 2.88E-18 | 2.13E-17 |
| B3GALT4 | 12.174569 | 4.5531461 | -1.418935 | 4.06E-23 | 1.44E-21 |
| EXTL1 | 0.1102321 | 0.0379008 | -1.540246 | 1.87E-13 | 7.18E-13 |
| ST6GALNAC1 | 100.40328 | 34.269693 | -1.550801 | 3.84E-16 | 2.00E-15 |
| GALNT17 | 0.9379081 | 0.3065111 | -1.613507 | 2.44E-11 | 6.87E-11 |
| PIGZ | 31.409007 | 9.7739601 | -1.684163 | 1.37E-16 | 8.11E-16 |
| GCNT4 | 1.2841992 | 0.3100824 | -2.050146 | 6.72E-19 | 5.41E-18 |
| GCNT3 | 65.384597 | 15.754766 | -2.053163 | 2.84E-20 | 3.14E-19 |
| GALNT15 | 1.9085806 | 0.427085 | -2.159905 | 1.91E-17 | 1.25E-16 |
| B3GNT6 | 21.461509 | 4.208447 | -2.350392 | 1.36E-20 | 2.00E-19 |
| ST8SIA3 | 0.0523908 | 0.009242 | -2.503045 | 1.80E-23 | 7.96E-22 |
| GALNT16 | 0.5864448 | 0.1021116 | -2.521848 | 5.38E-21 | 9.52E-20 |
| FUT9 | 0.0207591 | 0.0035253 | -2.557926 | 1.51E-19 | 1.58E-18 |
| CHST9 | 0.0559402 | 0.0094452 | -2.566228 | 5.36E-25 | 9.49E-23 |
| CHST5 | 6.634018 | 1.0973954 | -2.595799 | 3.65E-19 | 3.23E-18 |
| ST6GALNAC6 | 39.208008 | 6.314161 | -2.634485 | 1.95E-21 | 3.83E-20 |
| B3GALT5 | 12.530079 | 1.9103415 | -2.713493 | 1.92E-19 | 1.79E-18 |
| B3GNT7 | 75.979314 | 8.3345249 | -3.188435 | 1.79E-19 | 1.76E-18 |
| B3GALT1 | 3.0120143 | 0.3272911 | -3.202083 | 1.48E-22 | 4.36E-21 |
| GCNT2 | 2.660632 | 0.2119838 | -3.649743 | 1.18E-24 | 1.04E-22 |
| B4GALNT2 | 21.51408 | 1.1740161 | -4.195757 | 2.93E-22 | 7.41E-21 |

**Supplementary Table 2.** Glycosyltransferase-related differentially expressed genes between COAD and normal.

|  | LINC02381 | | AC002310.1 | | ZEB1-AS1 | | AC020558.2 | | AC105219.1 | | MIR210HG | | AC009237.14 | |
| --- | --- | --- | --- | --- | --- | --- | --- | --- | --- | --- | --- | --- | --- | --- |
|  | r | p | r | p | r | p | r | p | r | p | r | p | r | p |
| TNFRSF4 | **0.369** | **<0.001** | -0.006 | 0.911 | -0.011 | 0.833 | 0.049 | 0.342 | -0.041 | 0.430 | **0.211** | **<0.001** | -0.039 | 0.445 |
| CD200R1 | **0.279** | **<0.001** | **0.119** | **0.021** | **0.276** | **<0.001** | -0.074 | 0.153 | **-0.115** | **0.025** | **-0.109** | **0.034** | -0.003 | 0.951 |
| ARG1 | 0.042 | 0.413 | **0.122** | **0.018** | **0.248** | **<0.001** | **0.131** | **0.011** | -0.015 | 0.775 | 0.031 | 0.549 | -0.088 | 0.086 |
| CD160 | 0.096 | 0.063 | 0.024 | 0.642 | **0.213** | **<0.001** | **0.179** | **0.001** | -0.063 | 0.224 | -0.008 | 0.877 | **-0.105** | **0.040** |
| CXCL8 | **0.145** | **0.005** | 0.042 | 0.412 | -0.068 | 0.184 | **-0.180** | **0.000** | **-0.150** | **0.003** | 0.034 | 0.509 | **-0.132** | **0.010** |
| ADORA2A | **0.359** | **<0.001** | 0.037 | 0.467 | **0.192** | **0.000** | **0.266** | **<0.001** | -0.006 | 0.903 | 0.010 | 0.849 | 0.003 | 0.957 |
| IL4 | 0.059 | 0.251 | 0.044 | 0.398 | **0.113** | **0.028** | 0.032 | 0.540 | 0.077 | 0.134 | -0.002 | 0.963 | 0.011 | 0.829 |
| VEGFA | -0.091 | 0.077 | -0.050 | 0.333 | 0.022 | 0.673 | **0.344** | **<0.001** | 0.076 | 0.139 | **0.270** | **<0.001** | 0.093 | 0.070 |
| NOS2 | **-0.129** | **0.012** | **-0.150** | **0.003** | **-0.192** | **0.000** | 0.035 | 0.497 | 0.013 | 0.798 | **0.242** | **<0.001** | **-0.262** | **<0.001** |
| TNFRSF25 | -0.035 | 0.502 | 0.003 | 0.955 | -0.069 | 0.183 | **0.431** | **<0.001** | **0.291** | **<0.001** | **0.190** | **0.000** | **0.113** | **0.028** |
| TNFRSF14 | -0.072 | 0.161 | -0.050 | 0.329 | -0.045 | 0.382 | **0.256** | **<0.001** | 0.075 | 0.143 | **0.294** | **<0.001** | **-0.155** | **0.003** |
| EZH2 | **-0.212** | **<0.001** | 0.087 | 0.090 | -0.011 | 0.831 | 0.063 | 0.223 | 0.007 | 0.894 | -0.027 | 0.601 | -0.060 | 0.240 |
| TBX2 | **0.244** | **<0.001** | -0.073 | 0.158 | -0.035 | 0.494 | **0.145** | **0.005** | 0.043 | 0.402 | 0.069 | 0.177 | 0.094 | 0.068 |
| VSIR | **0.385** | **<0.001** | 0.010 | 0.843 | 0.075 | 0.146 | -0.041 | 0.425 | **-0.142** | **0.006** | **0.142** | **0.006** | -0.043 | 0.402 |
| TGFB1 | **0.425** | **<0.001** | 0.069 | 0.182 | **0.173** | **0.001** | -0.033 | 0.517 | -0.071 | 0.165 | **0.159** | **0.002** | -0.095 | 0.066 |
| HHLA2 | **-0.150** | **0.003** | **-0.122** | **0.018** | **-0.199** | **<0.001** | **-0.154** | **0.003** | -0.003 | 0.960 | 0.031 | 0.543 | -0.059 | 0.254 |
| NOS3 | **0.206** | **<0.001** | -0.081 | 0.117 | 0.009 | 0.858 | **0.118** | **0.022** | 0.078 | 0.127 | 0.047 | 0.362 | **0.125** | **0.015** |
| TNFSF14 | **0.469** | **<0.001** | 0.012 | 0.819 | **0.134** | **0.009** | 0.039 | 0.445 | -0.086 | 0.095 | 0.096 | 0.062 | **-0.151** | **0.003** |

**Supplementary Table3.** The correlation between immune-related genes and 7 prognostic GT-related lncRNA.


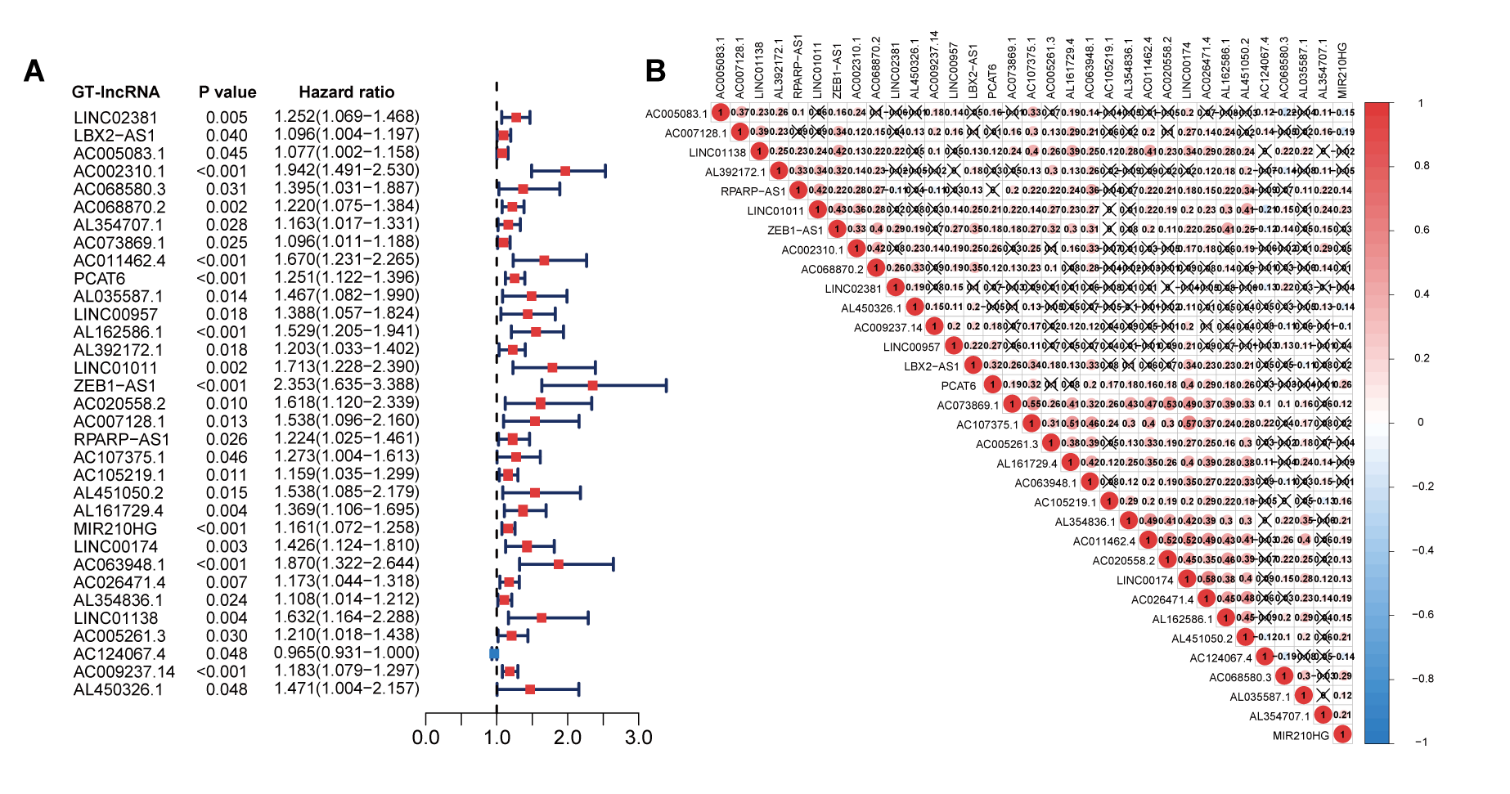


**Supplementary Figure 1.** Identification of 33 GT-related lncRNAs with prognosis value and correlation analysis of these lncRNAs. **(A)** Forest plot of 33 prognostic GT-related lncRNAs. (B) Correlation results of 33 prognostic GT-related lncRNAs. The red point represents a positive correlation, while the blue point represents negative correlation.


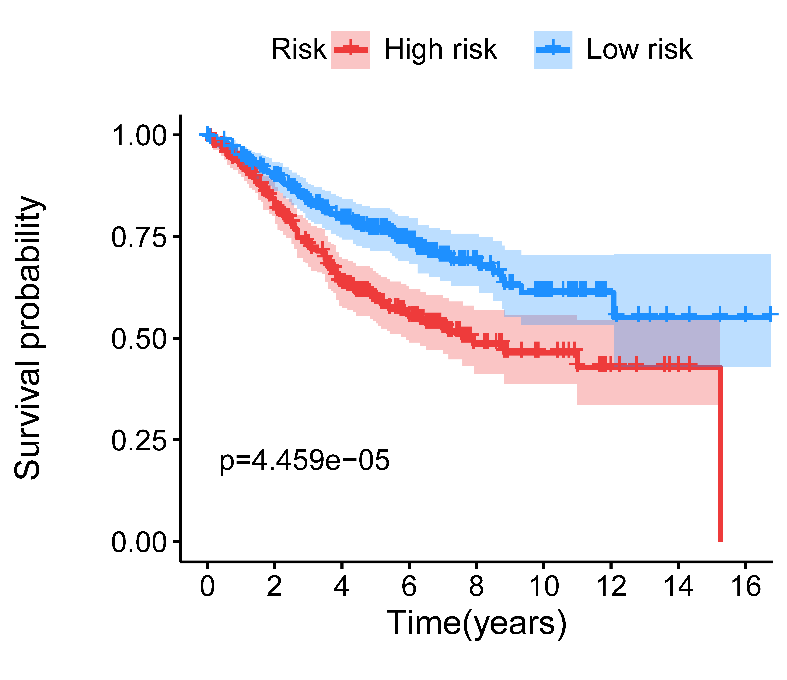


**Supplementary Figure 2.** Kaplan-Meier curve showing the significantly survival difference between high- and low-risk groups from the GEO dataset.


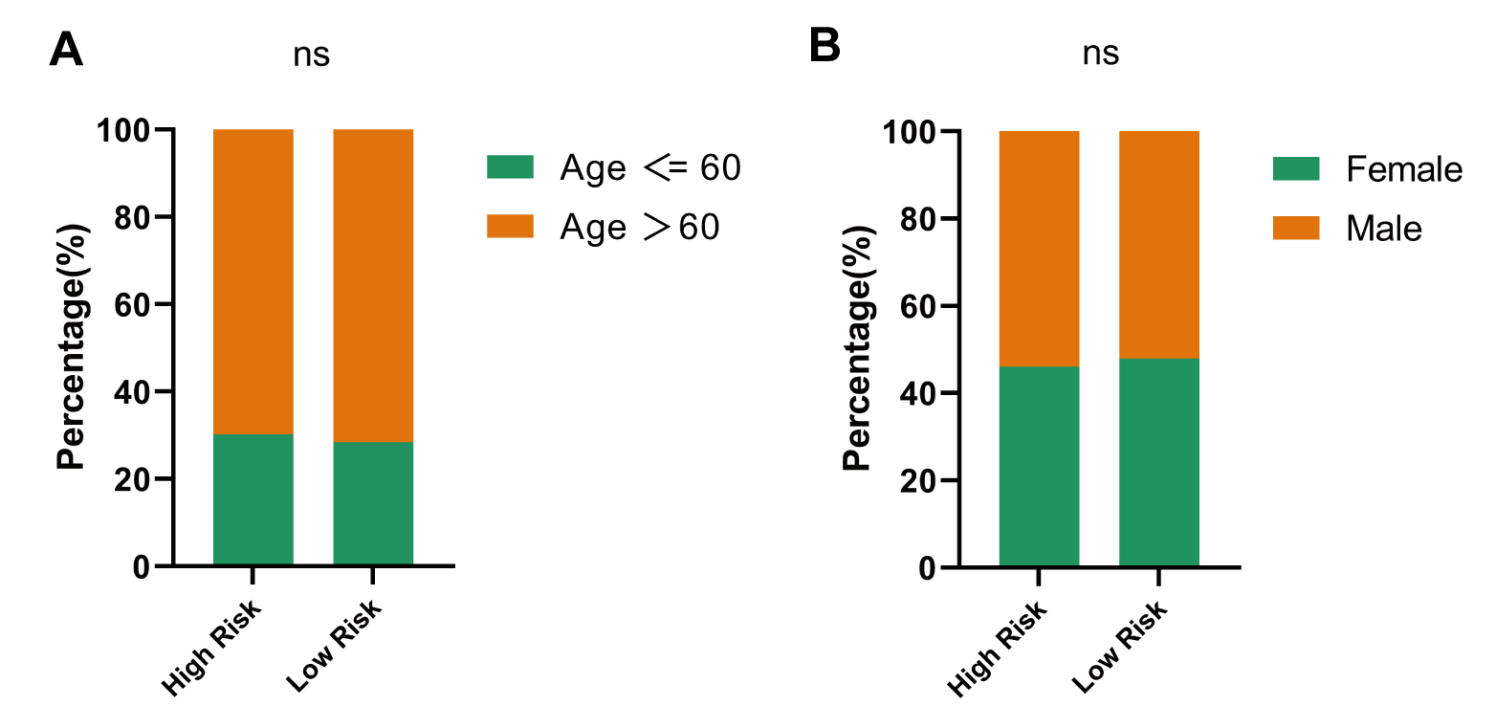


**Supplementary Figure 3.** The high- and low-risk groups showed no significant differences in age**(A)** and gender subgroups**(B)**.


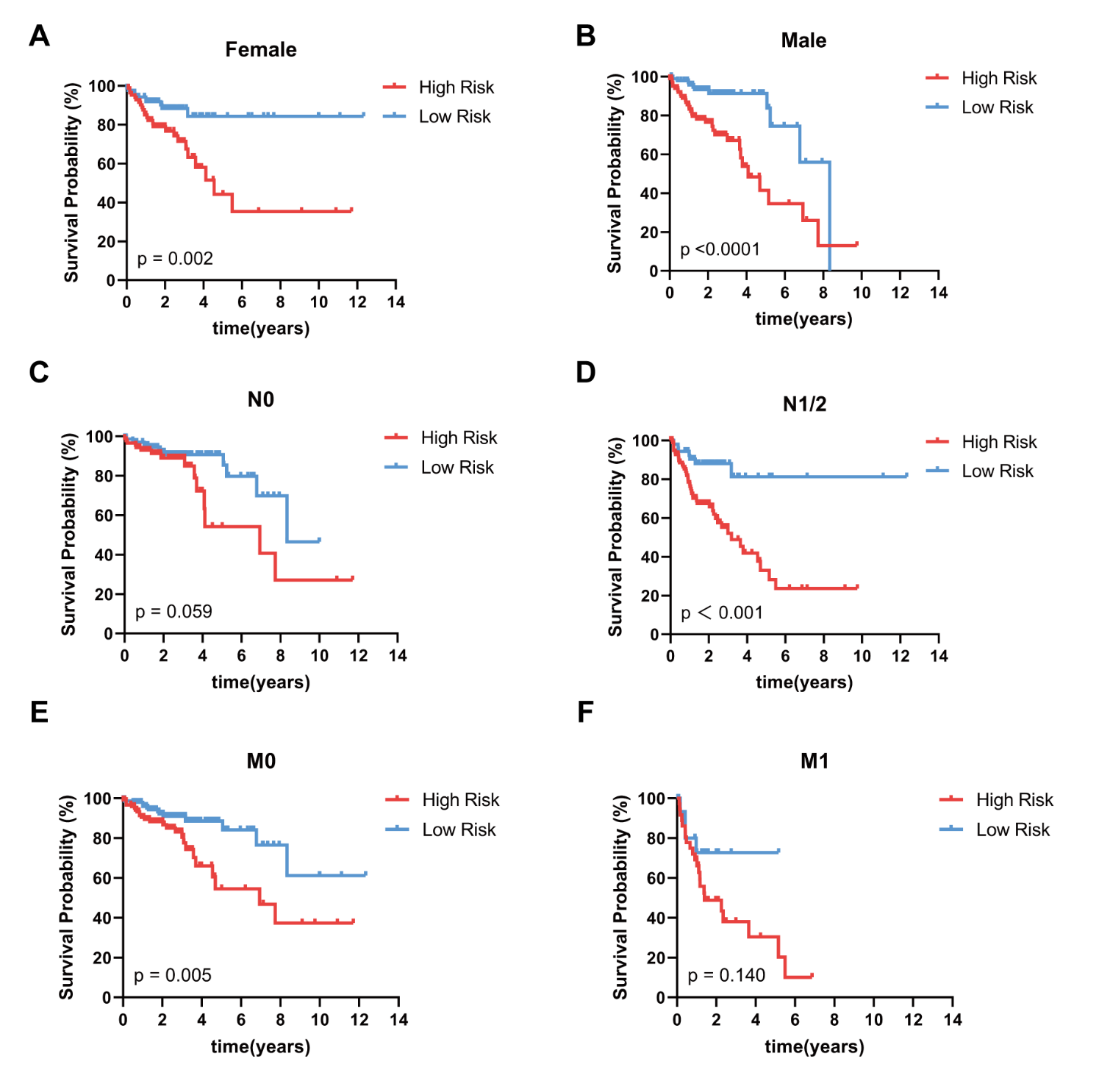


**Supplementary Figure 4.** The Kaplan-Meier curve shows the prognostic value of the risk model for COAD patients categorized by gender **(A, B)**, N stage **(C, D)**, and M stage **(E, F)**.
